# Social–Emotional Profiles of Preschool Children: An Investigation of Demographic Disparities and Intersectionality

**DOI:** 10.3390/ijerph21081100

**Published:** 2024-08-20

**Authors:** Chin-Chih Chen, Yaoying Xu, Jennifer LoCasale-Crouch, Yuyan Xia, Kathleen Rudasill, Lindai Xie, Karli Johansen, Jeen Joy, Jennifer Askue-Collins

**Affiliations:** 1 Department of Counseling and Special Education, School of Education, Virginia Commonwealth University, Richmond, VA 23284, USA; ccchen@vcu.edu (C.-C.C.); locasalecrj@vcu.edu (J.L.-C.); kmrudasill@vcu.edu (K.R.); xiel3@vcu.edu (L.X.); zilberfarbka@vcu.edu (K.J.); joyj2@vcu.edu (J.J.); askuecollij@vcu.edu (J.A.-C.); 2 Department of Physical Therapy, College of Health Sciences, University of Kentucky, Lexington, KY 40506, USA; yuyan.xia@uky.edu

**Keywords:** social–emotional profile, preschool-aged children, Head Start

## Abstract

This study aims to enhance our understanding of the diverse nature of social–emotional development and explore the demographic disparities and intersectionality of social determinants among children, with an emphasis on underserved populations of children in low-resource environments. Young children living in low-income families are exposed to a wide array of social and systemic risks that increase the propensity for poor learning and social–emotional development. Using data from the Head Start Family and Childhood Experiences Survey (FACES, this study focuses on the social–emotional development of a nationally representative sample of young children enrolled in the Head Start program (*n* = 1921, 50.18% male). Employing a person-centered approach, we assessed teacher-rated social–emotional competence, including approach to learning, social cooperation, aggression, hyperactivity, and anxiety/depression/withdrawal, to classify young children’s social–emotional development. This study identified four distinct social–emotional profiles—Adaptive, Average, Moderate Risk, and High Risk—through latent profile analysis. Furthermore, multinomial regression analysis revealed demographic disparities within each social–emotional profile, and significant intersectionality was found between race/ethnicity, age, and disability status in the social–emotional profiles. This research provides valuable insights for better supporting each child’s unique needs.

## 1. Introduction

Young children’s social–emotional skills are critical drivers of both school readiness and their future outcomes [1,2,3,4,5,6]. Approximately 20% of children, however, show significant social–emotional challenges at the start of school [7]. This percent is higher for racially minoritized children, children who are dual language learners (DLL), and those identified with a disability [3,8,9].Prior work has often treated social–emotional skills as a single construct, yet that approach does not reflect the varying constellation and combination of these skills that form individual patterns. Further, we have limited knowledge about how children with distinct characteristics, such as children from diverse racial/ethnic backgrounds, children with disabilities/developmental delays, or children of different gender or age might respond differently to instruction and how these characteristics influence their social–emotional development. Notably, there is a dearth of studies exploring the intersectionality of these attributes.

Understanding children’s unique social–emotional profiles is important so interventions and supports can be more targeted. Thus, this study aims to (1) characterize patterns in young children’s social–emotional skills among Head Start (The U.S. Department of Health and Human Services (HHS) Poverty Guidelines are used to determine income eligibility for participation in Head Start and Early Head Start programs. Children from birth to age five who are from families with income below the poverty guideline are considered from “low-income” families and are eligible for Head Start/Early Head Start programs.) (HS) children using a person-oriented approach; (2) identify disparities in the social–emotional development of HS children based on demographics such as gender, race/ethnicity, disabilities, and poverty; and (3) investigate the intersectionality of these social demographics. Findings will enable a more comprehensive understanding of young children’s social–emotional skills and the associated characteristics and experiences that intersect with them.

### 1.1. Young Children’s Social–Emotional Skills and Future Success

Social–emotional development encompasses the growth of skills such as comprehension of alternative viewpoints, peer and adult cooperation, emotional regulation, self-control, and problem-solving skills. Social–emotional skills are critical for school readiness [4]. At kindergarten entry, children are faced with heightened social and academic demands for well-regulated and goal-directed activity, including behavioral inhibition, compliance with rules, and the capacity to initiate and sustain positive interpersonal relationships with teachers and peers [10,11]. Indeed, social–emotional skills are consistent predictors of high school outcomes, predicting academic achievement, dropout risk, school connectedness, substance use, physical activity, and weight status in students [3,4,12,13,14]. In a recent study, Ricciardi and colleagues [15] found that social–emotional skills at age 5 predicted academic achievement, over and above the impact of pre-academic school readiness skills. Further, teachers identify behavioral issues as the main adjustment concern for children in kindergarten [16]. Children experience many heightened expectations while entering kindergarten, which can cause behavioral difficulties [10]. Social–emotional skills, thus, provide the foundational support for effective school engagement as they prepare children to follow classroom rules, cope actively with learning challenges, and relate to teachers and peers [14].

Currently, 20% of US children nationally are considered “not on track” with their social and emotional skills at the start of kindergarten [7] (, with the rate even higher for children from low-resourced families [14]. Additionally, a wide body of research indicates that there are significant gaps in children’s social–emotional skills across racial/ethnic lines [17] and disability status [18] at the start of kindergarten. Several studies have shown that children facing early emotional or behavioral challenges tend to perform poorly in language and numeracy [2,3,12,13], and evidence suggests that children who enter kindergarten behind their peers rarely catch up [19,20,21]. Thus, despite recognizing the documented impact of social–emotional skills on children’s early social and academic success, limited research has investigated the diversity of children’s social–emotional profiles and demographic disparities, particularly among marginalized, underserved young children in low-resourced communities.

### 1.2. A Nuanced Approach to Examining Young Children’s Social–Emotional Skills

Despite the significant contributions of young children’s social–emotional skills to school readiness and future success, most studies have focused on school-aged students [22,23,24,25]. A growing body of research, though, is now examining young children’s social–emotional skills. For example, Williams et al. [6] examined self-regulation, and Vogan et al. [26] focused on executive functioning as they relate to young children’s social–emotional skills. Thus, early socio-emotional skills are an area in need of further investigation.

While social–emotional development is crucial for early school success, understanding how these skills interact to form distinct child profiles remains under-researched. Previous variable-centered studies have examined average relationships between skills, but often overlook individual variations and the dynamic nature of development [27]. Person-oriented research, focusing on individual or subgroup development, can identify latent profiles based on diverse indicators (Bergman and Magnusson, 1997). This approach offers a more comprehensive and individualized understanding of children’s social–emotional development by examining how skills interact within individuals and how individuals cluster based on their skill profiles [27].

Recognizing the interconnectedness of a host of children’s social–emotional skills, some studies have begun to examine these skills together using a person-centered approach [27,28] or a latent profile analysis [29,30,31] to identify children’s social–emotional profiles. For example, two profile analysis studies on school readiness [32,33] indicated that better social–emotional ability in the Pre-K phase can ensure a higher school readiness, which may affect preschoolers’ academic performance in subsequent years of schooling. Additionally, Denham et al. [27] conducted a profile analysis on preschool-aged children’s social–emotional profiles and categorized participants into three groups: Social–Emotional Risk, Competent—Social/Expressive, and Competent—Restrained. Profile approaches to understanding children’s socio-emotional skills appears as a potentially helpful direction in understanding the intersection of these related skills.

Further, Collie et al. [34] investigated how social–emotional profiles are linked to important educational outcomes. They found sociodemographic characteristics tended to be associated with social–emotional profiles and outcomes such as academic achievement. Girls and older students tended to fall within the prosocial profile, while male students tended to exhibit behaviors categorized as aggressive [34] Collie et al. [34] further identified that students within non-English language groups were more likely to be in anxious or aggressive profiles rather than prosocial. Higher neighborhood SES was also associated with a prosocial profile. Additionally, their identified profiles were associated with different levels of achievement when socio-educational characteristics and early academic achievement were controlled. The highest academic achievement was associated with the prosocial profile. Thus, while more investigation in this area is needed, these early socio-emotional profiles seem to be connected to both other critical developmental domains as well as individual child characteristics.

### 1.3. Intersectionality of Young Children’s Identity and Learning Experiences

Some of the literature has examined the intersectionality of various identities among preschool-aged children that are related to their social–emotional skills [33,34,35] For example, social–emotional patterns of preschool-aged children who are dual language learners (DLL) suggest that school readiness profiles with language impairment are associated with the quality of the children’s classroom experience [32] Additionally, children who were rated higher on social skills and lower on behavioral concerns by their preschool teachers had greater English language skills compared to those who were rated lower on social skills and higher on behavioral concerns [36]. Social–emotional patterns of preschool children with a disability, specifically autism, show results of higher anxiety and depression [37]. Additionally, Tavassolie et al. [33] identified six school readiness profiles using a person-centered approach and ratings of school and home behavior with a sample of 43,044 (56.9% Latinx, 36.2% Black, and 6.9% White) four-year-old children, and found demographic characteristics were associated with profile membership. For example, girls tended to have profiles of pre-academic strength and positive behavior. Low-income children had a greater likelihood of being in a profile characterized by strong school and average home behavior rather than the profile of average school and strong home behavior. The overall poor school readiness profile was more likely to include children who were Black, DLL, have special needs, or in a low-income category. No significant differences were found between Latinx and White profile membership. Examining the intersectionality of children’s identity and their socio-emotional skills profile, appears warranted. Our approach adopts an intersectional lens, recognizing that children’s identities are not singular or isolated. Instead, we examine how social demographics—such as race, gender, and poverty status—intersect and shape their socio-emotional development. This approach moves beyond analyzing these factors in isolation and acknowledges the complex interplay of identities in children’s lives.

### 1.4. The Current Study

In sum, although some demographic factors such as gender, race/ethnicity, socioeconomic status (SES), or disability [23,33] have been explored in association with young children’s social–emotional development, the intersectionality of these social demographics with profiles of children’s socio-emotional skills have been less studied. Due to the limited research on preschoolers’ social–emotional profiles and its potential impact on children’s school readiness, the present study intends to analyze the social–emotional profiles of preschool-aged HS children. In the current study, we further explore demographic disparities and intersectionality of social demographics.

To address the gaps identified, in this study, we will deeply examine young children’s social–emotional development using data from the Head Start Family and Childhood Experiences Survey (FACES, 2014; United States Department of Health and Human Services). The FACES is a longitudinal, multistage study that includes a nationally representative sample of young children from low-income families. The following research questions are addressed.

1. How can the social–emotional profiles of young children participating in HS programs be characterized?
2. What is the predictive relationship between children’s demographic characteristics and their social–emotional profiles?
3. How do children’s different demographic factors intersect in relation to their social–emotional development?

We hypothesize that there will be heterogeneity in the social–emotional profiles of preschool children in HS programs, with distinct groups emerging based on varying levels of social–emotional competence. Additionally, we propose that demographic characteristics, such as gender, race/ethnicity, age, and disability status, will be associated with membership in different social–emotional profiles. Lastly, we anticipate that the relationship between demographic characteristics and social–emotional profiles will be moderated by other demographic factors, indicating intersectionality in social–emotional development.

## 2. Methods

### 2.1. Data Source, Research Design, and Participants

The data in this study were derived from the Family and Child Experiences Survey 2014 (FACES 2014; United States Department of Health and Human Services). FACES 2014 utilized a complex sample design, which included a nationally representative sample of over 2000 children and their families participating in HS programs during the 2014–2015 HS year. The sampling process involved a multistage probability sampling, with stratification based on the HS program. FACES 2014 implemented the systematic sampling approach of probability proportional to size (PPS) at the first two stages (HS programs and center within HS programs). This method involved selecting every nth case after a random starting point, effectively reducing the risk of selection bias. Subsequent to this initial stage, an equal number of classrooms (*n* = 2) were sampled within a selected center, and for each selected classroom, an equal number of children (*n* = 12) were chosen with equal probability at the final two stages. The goal was to ensure that each classroom and child received nearly equal representation in the study.

By utilizing various sources and methodologies, FACES 2014 offered researchers and policymakers valuable insights into children’s early education experiences, development, and learning progress over a period of time. The data collection process involved parent surveys, teacher ratings/surveys, and direct child assessments. FACES gathered information from parents to gain crucial perspectives. It also sought input from children’s teachers to assess their approaches to learning, cooperative behaviors in the classroom, and any problem behaviors.

The current study included 1921 children drawn from FACES 2014. The participating children were almost evenly split between children identified as male (50.18%) and female (49.82%). The ethnic composition of the sample was diverse, with 41.40% identifying as Hispanic, 25.70% as Black or African American, 23.72% as White, and 9.18% belonging to other racial/ethnic groups. Nearly 12% of the HS children in the study were identified with disabilities. Moreover, nearly 68% of the HS children lived at or below the federal poverty threshold (FPL). The average age of the children was 48.34 months (note: percentages listed above were weighted to ensure accuracy).

### 2.2. Measures

The FACES instruments provided valid and reliable information on the social–emotional competence of low-income preschool children from diverse cultural and linguistic backgrounds and children’s progress over the 2014–15 HS year. The primary measures of interest are described below.

#### 2.2.1. Social–Emotional Competence

This variable was evaluated through a teacher report of children’s positive and problematic behaviors, encompassing three significant domains: approach to learning, social cooperation, and problem behaviors (i.e., aggressive, hyperactive, and anxious/withdrawn behaviors) in the fall 2014 and spring of 2015 (note: this study used the data collected in the spring of 2015 only). These domains are both theoretically and empirically linked to the multi-faceted construct of social–emotional development.

#### 2.2.2. Approaches to Learning

This variable was assessed through a teacher report derived from the Approaches to Learning Scale sourced from the Early Childhood Longitudinal Study(ECLS-K) [38] Teachers rated six items related to a child’s motivation, attention, organization, persistence, and independence in learning using a 4-point Likert scale, ranging from “never” to “very often”. A composite score was generated and standardized, with higher scores indicating a more frequent approach to learning. The internal consistency reliability (alpha) coefficients ranged from 0.92 to 0.93 from fall 2014 through spring 2015.

#### 2.2.3. Social Cooperation

Teachers assessed children’s engagement in cooperative classroom behaviors using a 3-point Likert scale, ranging from “never” to “very often” to score 12 items (e.g., following the teacher’s directions, helping put things away, complimenting classmates, and following rules when playing games). These items were sourced from both the Personal Maturity Scale [39] and the Social Skills Rating System (SSRS) [40]. Subsequently, a composite score was created and standardized, with higher scores indicating more frequent cooperative classroom behaviors. The internal consistency reliability (alpha) coefficients of the total cooperative behavior summary score ranged from 0.88 to 0.89 from fall 2014 through spring 2015.

#### 2.2.4. Problem Behaviors

Children’s problem behaviors were measured using items derived from an abbreviated adaptation of the Personal Maturity Scale [39] (Entwisle et al., 1987) and the Behavior Problems Index (BPI) [40,41]. Teachers used a 3-point Likert scale, ranging from “not true” to “very true or often true”, to indicate the extent to which children exhibit certain characteristics of problem behaviors. There are 4 items used to assess child aggressive behaviors (e.g., hitting/fighting with others, disobeying rules or requests, and disrupting ongoing activities), 3 items to assess child hyperactive behaviors (e.g., restlessness, constant fidgeting, inability to sit still, and being easily distracted by room activities), and 6 items to assess child anxious or depressed and withdrawn behaviors (e.g., unhappiness, withdrawal, nervousness, high strung, or tension). A composite average score was derived from each subscale and standardized, with higher scores indicating a higher frequency of problem behaviors. The internal consistency reliability (alpha) coefficients of the total problem behavior summary score ranged from 0.88 to 0.89 from fall 2014 through spring 2015. 

#### 2.2.5. Child Demographics

We included gender (male = 1, female = 0), race/ethnicity (White, Black or African American, Hispanic, or “Other”), age, disability status, and poverty status (at or below the federal poverty level [FPL]).

### 2.3. Analytical Plan

We explored the potential mechanisms of missingness for the variables used, starting with Little’s MCAR test [42] to test the assumption that the data were missing completely at random. The assumption was met (*Chi*-Square = 8.937, DF = 6, Sig. = 0.177). To address any potential bias introduced by missing data, we employed full information maximum likelihood (FIML) estimation [43].

We employed latent profile analysis (LPA, see Table 1) to identify distinct subgroups of HS children based on their social–emotional patterns/subtypes, using a defined set of five variables (i.e., approach to learning, social cooperation, and problem behaviors—aggressive, hyperactive, and anxious/withdrawn behaviors) [44] LPA, known as a person-centered approach, allows for us to classify students who are relatively homogeneous within their subgroups and heterogeneous between different subgroups in terms of their social–emotional competence. Multiple LPA iterations were performed using Mplus to determine the optimal number of social–emotional profiles among HS children in spring 2015. In LPA, the indicator variances were constrained to be equal across profiles. The estimation process utilized the default settings in Mplus, with 20 random starts and 200 final stage optimizations to ensure the identification of the global maximum likelihood solution. To further ensure accuracy, we ran the model with at least twice the number of random starts. In LPA, the indicator variances were constrained to be equal across profiles. This assumption is based on research suggesting that social–emotional skills develop along a common trajectory in early childhood, with individual differences reflecting variations in the rate or intensity of development, rather than fundamentally different [27]. Various fit indices, including log likelihood, Akaike information criterion (AIC), Bayesian information criterion (BIC), and adjusted BIC [45], were employed to ascertain the optimal number of latent profiles. Additionally, the adjusted Lo–Mendell–Rubin likelihood ratio test (ALMR) [46] and the Vuong–Lo–Mendell–Rubin likelihood ratio test (VLMR) were used to compare the relative model fit, with a *p*-value less than 0.05 indicating support for the k-class model over the k − 1 class model. Furthermore, we evaluated the classification accuracy of the number of classes using entropy, which ranges from 0 to 1, and higher values reflect better accuracy in classifying the children into their respective subgroups.

Using the Mixture modeling-3 step method [47,48], we explored the association between demographic characteristics and children’s distinct social–emotional profiles (refer to Table 2). Additionally, we investigated the intersection of various demographic factors in influencing children’s social–emotional development (refer to Table 3). In our intersectionality analysis, we examined whether the social–emotional profiles of HS children from diverse racial/ethnic backgrounds varied based on gender (model 1), age (model 2), disability (model 3), and poverty status (model 4) while controlling for other variables. Taylor Series methods were employed to ensure unbiased variance estimation and account for the complex sample design. All estimates and standard errors included in the data tables are based on weighted data, providing accurate representations of national estimates.

## 3. Results

### 3.1. Heterogeneity of Social–Emotional Profiles

As shown in Table 1, we identified five viable models in the initial model testing phase because the six-class model did not replicate the best log likelihood value. With an increased number of classes, all model fits exhibited significant improvement, with decreasing values of BIC, AIC, and SABIC. Additionally, entropy values of 0.80 or higher provided supportive evidence that the classification of individuals within the model occurred with minimal uncertainty. All five models under consideration successfully met this criterion. We plotted the log likelihood values and searched for a bend or “elbow” to identify the point at which the gain from model improvement began to diminish relative to the additional parameters estimated [48]. Following this criterion, our study identified an “elbow” in the log likelihood values curve thus resulting in the four-class model as the final model (log likelihood = −10,228.14, AIC = 20,512.28, BIC = 20,666.90, SABIC = 20,577.94, entropy = 0.88).

Four distinct social–emotional profiles—labeled as Adaptive, Average, Moderate Risk, and High Risk—were identified. Table 2 presents the mean scores and standard errors for each social–emotional profile of HS children, using ±0.50 as a cutoff point to describe scores above or below the average of teacher-rated child approach to learning, social cooperation, aggressive, hyperactive, and anxious or depressed and withdrawal behaviors.

**Figure 1:**
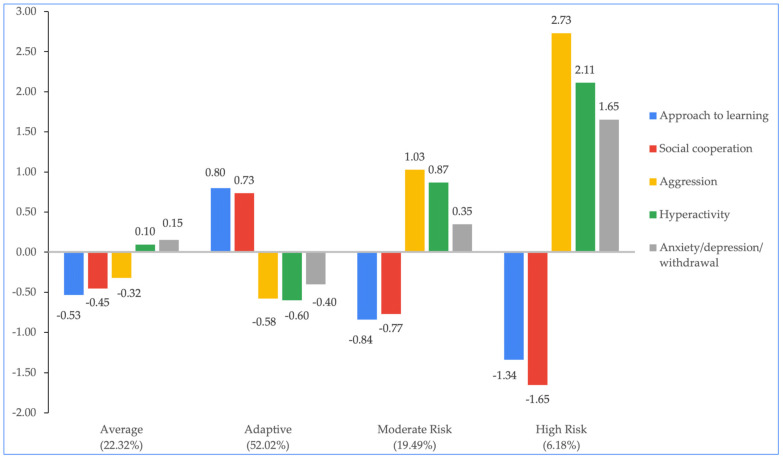
Characteristics of HS subgroups of distinct social–emotional profiles. Note: we use ±0.50 as a cutoff point to describe the SE scores above or below the average.

The social–emotional profiles of HS children were characterized as follows: (a) Average (22.32%): exhibiting average scores on approach to learning, social cooperation, aggressive, hyperactive, and anxious or depressed and withdrawn behaviors. (b) Adaptive (52.02%): displaying above-average scores on approach to learning and social cooperation, below-average scores on aggressive and hyperactive behaviors, and average scores on anxious or depressed and withdrawn behaviors. (c) Moderate Risk (19.49%): showing below-average scores on approach to learning and social cooperation, above-average scores on aggressive and hyperactive behaviors, and average scores on anxious or depressed and withdrawn behaviors. (d) High Risk (6.18%): indicating below-average scores on approach to learning and social cooperation, above-average scores on aggressive and hyperactive behaviors, and above-average scores on anxious or depressed and withdrawn behaviors (See Figure 1).

### 3.2. Demographic Disparities

As shown in Table 2, compared to girls, boys were 1.84 times more likely to be classified as Average (*β* = 0.61; *p* < 0.01), 3.29 times more likely to be classified as Moderate Risk (*β* = 1.19; *p* < 0.01), and 4.62 times more likely to be classified as Higher Risk (*β* = 1.53; *p* < 0.01) rather than Adaptive. When compared to White children, Black children were 2.06 times more likely to be classified as Average (*β* = 0.72; *p* < 0.01), 1.72 times more likely to be classified as Moderate Risk (*β* = 0.54; *p* < 0.05), and 2.69 times more likely to be classified as Higher Risk (*β* = 0.99; *p* < 0.05) rather than Adaptive. Hispanic children were 0.51 times more likely to be classified as Moderate Risk rather than Adaptive (indicating a 49% lower risk; *β* = −0.68; *p* < 0.05). Older children were 0.94 times more likely to be classified as Average, Moderate Risk, and High Risk rather than Adaptive (*β* = −0.06; *p* < 0.01). Furthermore, in comparison to children without disabilities, children with disabilities were 1.66 times more likely to be classified as Moderate Risk (*β* = 0.51; *p* < 0.05) and 4.55 times more likely to be classified as Higher Risk (*β* = 1.51; *p* < 0.01) rather than Adaptive. Regarding children’s family income, the multinomial logits for those living below or above the FPL (federal poverty line) did not show significant differences, assuming all other variables in the model remained constant. In other words, there was no significant difference in the classification of social–emotional profiles based on their poverty status.

### 3.3. Intersectionality

Table 3 presents the results of the intersectionality analysis, which examined whether the social–emotional profiles of HS children from diverse racial/ethnic backgrounds differed based on their gender (model 1), age (model 2), disability (model 3), and poverty status (model 4), while controlling for other variables. When comparing children with different racial/ethnic backgrounds to White children, the multinomial logits predicting distinct social–emotional profiles did not vary significantly by gender. In terms of the intersectionality between racial/ethnic backgrounds and age, compared to White children, Black and children identified as being in other racial groups were more likely to be classified as Moderate Risk than Adaptive children (*β* = 0.06; 0.16, *p* < 0.05). Regarding the intersectionality between racial/ethnic backgrounds and disability status, the marginal effect indicated that Hispanic children were more likely to be classified as Moderate Risk than Adaptive when they had disabilities (*β* = 1.45, *p* < 0.10). It is worth noting that the predictive relationship between race/ethnicity and children’s social–emotional profiles did not significantly differ by poverty status, assuming all other variables in the model remained constant. In other words, there was no significant difference in the classification of social–emotional profiles based on poverty status among children of Black, Hispanic, and children identified as being in other racial groups compared to White children.

## 4. Discussion

The goal of this study was to examine the social–emotional development profiles of HS children and how the profiles intersect with their sociodemographic characteristics. Three key findings emerged. First, four distinct social–emotional profiles of HS children were identified in this study. Additionally, children’s socio-emotional profiles were related in various ways to their sociodemographic characteristics. And, the uniqueness of HS children, both in their socio-emotional profiles and in their demographics, emerged when considering all these factors together. These findings are timely and informative for planning targeted interventions to prepare children with readiness skills, particularly their socio-emotional skills, prior to their entering kindergarten. These points will be further elaborated on below.

### 4.1. Heterogeneity of Social–Emotional Profiles

Through latent profile analyses, four distinct socio-emotional profiles emerged across HS children. Each profile presented a unique constellation of skills, with the majority of students falling into the Adaptive (52.02%) or Average (22.32%) profiles. This suggests that many children in HS, while experiencing risk such as low-income, exhibit well-developed socio-emotional skills. On the other hand, nearly 20% of children were in the Moderate Risk profile and 6% in the High Risk group. This points to a need for immediate attention for intervention because these emerging and high-risk factors may suggest significant social–emotional difficulties, which can contribute to falling further behind and contribute to achievement gaps at kindergarten entry and later school performance.

Another finding of the current study highlights the impact of children’s anxious, depressed, and withdrawn behaviors on their educational outcomes [34]. Shy or withdrawn children are particularly at risk of going unnoticed in the classroom [49,50]. These behaviors may not receive timely intervention unless they are accompanied by other indicators, such as a lack of social cooperation or engagement in learning. For instance, among the four social–emotional profiles, only the High Risk group suggests above-average anxious or depressed and withdrawn behaviors; children from the other three groups all received average scores on this variable, including the Adaptive group. While it is encouraging that the largest profile group may suggest positive social–emotional functioning in general, as evidenced by above-average scores on approach to learning and social cooperation, a closer examination needs to be taken to further identify factors related to their anxiety or depression and withdrawn behaviors, due to the average scores on these items. In other words, there might be some other demographics (such as child temperament, parent mental health, or home learning environment, [49] that could contribute to the average scores related to their anxious or depressed and withdrawal behaviors. This finding further suggests heterogeneity of HS children’s social–emotional development and learning within and between profiles. Early identification of children’s strengths (such as strong social cooperation) and weaknesses (such as being depressed or withdrawn) could inform teachers and caregivers with timely resources to develop effective interventions.

### 4.2. Children’s Socio-Emotional Profiles are Varied by Demographics

The current study identified significant relationships between the social–emotional profiles and children’s gender, race/ethnicity, age, and disability status. Specifically, boys are more likely than girls to be classified as Average, Moderate Risk, and High Risk than the Adaptive profile. This finding is consistent with Collie et al.’s (2019) study, which suggests that girls tended to fall within the prosocial profile while male students tended to exhibit behaviors categorized as aggressive. Additionally, the current study suggests older children are more likely to be classified as Adaptive also aligns with Collie et al.’s [9] study, which indicates older students tended to be classified as prosocial. This finding confirms the critical timing of preschool age prior to their entry to kindergarten, as the current study attempts to address. It is encouraging to see the positive pattern of developing adaptive skills with age growth so that preschool-aged children will be prepared with appropriate social–emotional skills and other related skills required for formal school.

The current study further suggests that Black children are more likely than White children to be classified as Average, Moderate Risk, and High Risk profiles compared to the Adaptive profile. The findings also suggest that children with disabilities are more likely than children without disabilities to be classified as Moderate Risk and High Risk compared to Adaptive. These demographic disparities confirm the need for examining the intersectionality of young children’s demographic characteristics and early schooling experiences related to their social–emotional learning [33,35,51].

### 4.3. Intersectionality of Race/Ethnicity and Other Demographics on Children’s Social–Emotional Profiles

In line with the recent literature examining the intersectionality of children’s various identities and their social–emotional skills as well as school readiness profiles [17,33,35], the current study closely examined the intersectionality of HS children’s racial/ethnic backgrounds and their gender, age, disability, and poverty status, respectively, on children’s social–emotional profiles. Findings indicated that gender was not a significant predictor of children’s social–emotional profiles when children from different racial/ethnic backgrounds were compared to White children. However, there was a significant intersectionality with age when children from different racial/ethnic backgrounds were compared to White children. Specifically, Black and children from other racial/ethnic groups are more likely to be classified as Moderate Risk than Adaptive, as discussed above. Furthermore, the older they are, the more likely they are classified as Moderate Risk. Longitudinal studies, then, need to identify additional factors that may impact children’s social–emotional profiles.

An interesting finding from the current study was the intersectionality with disability status specifically related to Hispanic children. While in general Hispanic children are less likely to be classified as Moderate Risk compared to Adaptive, when disability status was included in the relationship, it changed the direction. In other words, when Hispanic children have disabilities, they are more likely to be classified as Moderate Risk than Adaptive. This finding may partially explain the disproportionate representation of Hispanic children who receive special education services when they reach school age, possibly due to the lack of resources for Hispanic children with disabilities and their families, which could lead to either over-representation or under-representation in special education and related services.

Finally, unlike patterns observed in other studies, poverty status was not a significant predictor between race/ethnicity and children’s social–emotional profiles [33,34]. This may be due to HS programs serving predominantly children from low-income families, with over 68% of the HS children living at or below the federal poverty threshold (FPL), creating low or limited variability. Future studies can compare children from diverse racial/ethnic backgrounds and the intersectionality of SES at all levels.

## 5. Strengths and Limitations

Research on young children’s social–emotional development has recognized developmental and environmental factors through variable-based studies. This study contributes to the literature using data from the Head Start Family and Childhood Experiences Survey (FACES, 2014), which is a longitudinal, multistage study providing a nationally representative sample focusing on young children from low-income families. Through advanced data analyses, this large scale dataset provides a comprehensive profile of young children’s social–emotional development, particularly for those from marginalized backgrounds who have been underserved due to limited resources or other risk factors.

One limitation of this study was that the dataset that was conducted in the 2014–2015 academic year, which did not represent the most current data. We selected this dataset considering the longitudinal feature of the data to capture changes of children’s social–emotional profiles over time. The 2020 FACES data did not include classroom observations of children due to the interruption of COVID-19. Future research could employ similar research methods to updated data to further verify the identified variables and the intersectionality of factors influencing children’s social–emotional development.

## 6. Conclusions

Children participating in HS programs have heterogeneous social–emotional profiles, as identified in the current study. Understanding the varying factors associated with children’s social–emotional profiles can be helpful to bridge research, policy, and practice through the analyses of multilevel contextual factors. The significant demographic disparities of HS children’s social–emotional profiles further provide valuable information for effective interventions for children who are traditionally under-served from low-resourced communities. Yet, we should not view these contextual factors separately without close examinations of interactive relationships between race/ethnicity and other demographics such as gender, age, disability, and poverty. Future research may also need to examine subgroups such as DLLs further and the intersectionality of home language and children’s social–emotional development.

## Figures and Tables

**Table 1:** Latent profile analysis of HS children’s social–emotional profiles.

Classes	Log Likelihood	AIC	BIC	SABIC	LMR	LMRR	Entropy
1-class	−13,084.03	26,188.05	26,243.27	26,211.50	x	x	x
2-class	−11,210.47	22,452.94	22,541.29	22,490.46	0.00	0.00	0.90
3-class	−10,514.44	21,072.87	21,194.35	21,124.46	0.01	0.02	0.90
4-class	−10,228.14	20,512.28	20,666.90	20,577.94	0.16	0.17	0.88
5-class	−10,099.56	20,267.11	20,454.86	20,346.84	0.77	0.77	0.87
6-class	No convergence	-	-	-	-	-	-

**Table 2:** Demographic characteristics predictive of the HS children’s social–emotional profiles.

	Average vs. Adaptive			Moderate Risk vs. Adaptive			High Risk vs. Adaptive		
	*β*	Exp (*β*)	(95% Confidence Interval)	*β*	Exp (*β*)	(95% Confidence Interval)	*β*	Exp (*β*)	(95% Confidence Interval)
Male	0.61	1.84 **	(1.40–2.41)	1.19	3.29 **	(2.41–4.51)	1.53	4.62 **	(2.88–7.42)
Black	0.72	2.06 **	(1.35–3.16)	0.54	1.72 *	(1.06–2.80)	0.99	2.69 *	(1.19–6.09)
Hispanic	−0.07	0.93	(0.63–1.39)	−0.68	0.51 *	(0.30–0.87)	−0.61	0.54	(0.24–1.23)
Other	0.05	1.05	(0.64–1.74)	−0.72	0.49 ^+^	(0.22–1.05)	0.36	1.44	(0.77–2.67)
Age	−0.06	0.94 **	(0.92–0.97)	−0.06	0.94 **	(0.92–0.97)	−0.06	0.94 **	(0.90–0.98)
Disabilities	0.32	1.37	(0.82–2.29)	0.51	1.66 *	(1.04–2.63)	1.51	4.55 **	(2.05–10.09)
Poverty	0.11	1.12	(0.77–1.63)	0.15	1.16	(0.86–1.56)	0.34	1.41	(0.72–2.75)

Note: Adaptive profile as the reference group in the social–emotional profiles using female, White, non-disabled, and non-poverty status as the reference predictors; ^+^ *p* < 0.10; * *p* < 0.05; ** *p* < 0.01; *β* = coefficient; Exp (*β*) = relative risk ratio.

**Table 3:** Intersectionality of different demographics predicting the HS children’s social–emotional profiles.

	Average vs. Adaptive				Moderate Risk vs. Adaptive				High Risk vs. Adaptive			
	Model 1 *β*	Model 2 *β*	Model 3 *β*	Model 4 *β*	Model 1 *β*	Model 2 *β*	Model 3 *β*	Model 4 *β*	Model 1 *β*	Model 2 *β*	Model 3 *β*	Model 4 *β*
Male	0.48	0.61 **	0.61 **	0.62 **	0.99 **	1.21 **	1.19 **	1.19 **	1.69 **	1.54 **	1.52 **	1.53 **
Black	0.54 ^+^	0.52	0.77 **	0.70	0.36	−2.21	0.43 +	0.69	1.28 *	−0.64	0.72	0.89
Hispanic	−0.08	0.61	−0.07	0.16	−0.91 +	−1.99 *	−0.92 **	−0.77 **	−0.98	−2.07	−0.74	−0.20
Other	−0.01	−0.47	0.12	0.28	−0.68	−6.80 **	−0.96 *	−0.76	0.98	1.01	0.16	0.62
Age	−0.06 **	−0.05 +	−0.06 **	−0.06 **	−0.06 **	−0.09 *	−0.06 **	−0.06 **	−0.06 **	−0.08 *	−0.06 **	−0.06 **
Disabilities	0.32	0.32	0.36	0.32	0.51 *	0.52	−0.16	0.52 *	1.52 **	1.53 **	0.98	1.51 **
Poverty	0.11	0.11	0.12	0.28	0.14	0.14	0.14	0.20	0.32	0.36	0.31	0.53
Bla*Male	0.39				0.36				−0.30			
His*Male	0.06				0.38				0.48			
Oth*Male	0.13				−0.06				−0.87			
Bla*Age		0.00				0.06*				0.03		
His*Age		−0.01				0.03				0.03		
Oth*Age		0.01				0.12*				−0.02		
Bla*Disab			−0.47				0.49				0.81	
His*Disab			0.21				1.45 +				0.65	
Oth*Disab			−0.46				1.21				0.65	
Bla*Pov				0.01				−0.22				0.08
His* Pov				−0.34				0.11				−0.59
Oth*Pov				−0.37				0.07				−0.42

Note: Adaptive profile as the reference group in the social–emotional profiles using female, White, non-disabled, and non-poverty status as the reference predictors; ^+^ *p* < 0.10; * *p* < 0.05; ** *p* < 0.01; *β* = coefficient.

## Data Availability

Data analyzed in this study were from the Head Start Family and Child Experiences Survey (FACES) 2014. Users interested in obtaining these data must complete a Restricted Data Use Agreement.
